# Assessing the impact of demand response programs on the reliability of the Ghanian distribution network

**DOI:** 10.1371/journal.pone.0248012

**Published:** 2021-03-11

**Authors:** Ernestina M. Amewornu, Nnamdi I. Nwulu

**Affiliations:** Department of Electrical and Electronic Engineering Science, University of Johannesburg, Johannesburg, South Africa; University of Science and Technology of China, CHINA

## Abstract

The balancing of supplied energy to energy demand is often very challenging due to unstable power supply and demand load. This challenge causes the level of performance of distribution networks to be lower than expected. Research has however, shown the role of demand response (DR) on the performance of power networks. This work investigates the influence of DR, in the presence of incorporated renewable energy, on technical loss reduction, reliability, environment, energy saved and incentives paid to consumers with the help of PSAT and AIMMS software. Results from simulation have shown that the introduction of renewable energy into a Ghanaian distribution network coupled with implementing the proposed DR improves total energy supply by 9.8% at a corresponding operation cost reduction of 72.79%. The GHG and technical loss reduced by 27.26% and 10.09% respectively. The total energy saving is about 105kWh and 5,394.86kWh, for domestic and commercial loading profiles, respectively. Incentives received by consumers range between 45.14% and 58.55% more than that enjoyed, without renewable energy, by domestic and commercial consumers. The utility benefit also increased by 76.96% and 67.31% for domestic and commercial loads than that without renewable energy. Network reliability improves with implementation of DR. However, the reliability of a grid-connected network is better with a diesel generator only than with the integration of renewable energy. The power distribution companies, therefore, need to consider the implementation of incentive-based demand response program.

## 1. Introduction

One of the challenges associated with power distribution operation is ensuring a balance between electricity supply and load demand. The challenge is due to electricity demand increases beyond predicted levels. An approach to managing this power system challenge is adjusting the load supplied by implementing load shedding programs [1]. This approach involves distribution companies forcing some consumers to go off without the concern of the consumer. The involvement of consumers in load management will allow the consumers to decide on load (s) to turn off when the need arises.

An alternative approach to satisfying load demand is by encouraging end-users to curtail power. Electricity consumers can be involved in load management through the implementation of demand response (DR) programs. The performance of DR benefits both utility and consumers, depending on the contract between both parties [2–12]. The benefits associated with available options will influence stakeholders to be part of the DR program implementation.

The energy saved and financial gain to utility providers and consumers, associated with the influence of DR forms the bases of the assessment by [3–6, 11, 12]. The research by [3] evaluated the benefits of DR in a distribution network based on the tariff reduction according to energy curtailed. The power curtailed and tariff reduction level is influenced by bidding by both the consumer and the utility provider. The probability that the players will honour the agreement was base on analysis in simulated annealing (SA) Q- learning algorithm based on available records. The evaluation of DR benefits [4, 6] was based on a mandatory DR program, where incentives are paid to comply with the directive to curtail power and penalties paid for ignoring agreement to curtail power. Both researchers investigated the mandatory DR program effect through numerical analysis of the Iranian power network. During the examination, the economic indices of concern were electric energy consumption cost, consumer losses, and revenue loss to utility providers. The investigation also studied the DR program impact on peak load reduction, electrical energy consumption reduction, load factor and the peak to valley distance.

Additionally, [6] included in the assessment, the reliability at both supply and consumer end. This assessment was carried out through modelling and solving with the help of GAMS software. The reliability indices investigated were expected energy not supplied (EENS) and expected interruption cost (ECOST).

Other evaluations of the influence of DR on the reliability of distribution networks are [5, 7, 10] by considering of the benefit of the implementation of DR. The research by [5] measured the influence of DR on power network reliability by considering various power system indices. The indices include system average demand response frequency index (SADFI), customer average demand response frequency index (CADFI), system average demand response duration index (SADDI) and customer average demand response duration index (CADDI). The reliability assessment results were obtained from analytical and Monte Carlo simulation (MCS) techniques. As determined by [7], the influence of DR was based on the performance of components within a power system network. The concert was determined based on the time to failure (TTF), time to repair (TTR), time to isolate (TTI) and time to switch (TTS). The selection of consumers for DR program implementation depends on how often and frequent the consumer has already been deprived of power. The reliability indices considered by [7] include customer interruption (CI), customer minutes lost (CML), EENS and expected interruption cost (EIC).

The research by [7] also assessed the risk associated with implementing DR and compared the outcome with inter-trip systems. The evaluation of the risk associated with DR is according to sequential Monte Carlo simulation. The reliability evaluation by [10] was subject to the influence of DG at an optimal location and the implementation of DR. The optimal location of the DG was determined using an improved placement index while the reliability of a distribution network under DR was based on BPSO scan. The binary particle swarm optimization (BPSO) scan determines the failure rates of the network components. The evaluation of a distribution network reliability was based on the network components failure and repair times.

The research by [8, 9, 12], presents renewable energy influence on DR. programs. The benefits derived from implementing DR were measured [8, 12] in terms of load profile modification and incentive disbursed. The evaluation by [8] included power transfer from the grid and renewable energy sources. The DR program assessment [12] considered renewable energy, sited in the distribution network based on Genetic Algorithm and greenhouse gas emissions. Technical losses associated with integrating renewable energy into a Distribution network formed the bases for evaluating the impact of DR [9]. The results were derived from a simulationin MATLAB. The research [11] on the influence of DR in a network with multi-energy consumers was based on time of use. The benefit of DR, in this case, was also based on cost reduction, load profile and cost of energy purchased.

Electricity from renewable energy sources are unstable and could be the primary course of uncertainty in the available electrical power to end users depending on the penetration level. The uncertainty level considered in a project influences an electricity generation system’s operation cost and healthiness [13]. A reduction in risk factor associated with the uncertainty nature of renewable potentially contributes to enhancing the possibility of the self-healing a smart grid system [14]. The prediction of electrical power that can be generated from renewable energy sources is therefore very vital for the performance of a microgrid system.

The influence of DR in a distribution network has been assessed by the various researchers mentioned above. However, [3] evaluated the benefits of DR only on reducing tariff without explicit attention to the service of DR to utility providers, reliability and the influence of DG. Although [4, 6] determined the impact of DR on both stakeholder, the researchers overlooked the effect of combining DR and distributed generation (DG). Evaluation of DR contribution to the reliability of a power network in addition to the cost-benefit to stakeholders by [5–7, 10] is positive for decision making. However, the influence of renewable energy was omitted. The benefits of integrating DR into a power network [8, 9, 12] is determined in the presence of renewable energy. [8] considered the influence of electricity transfer from main grid but overlooked the influence of line losses and the impact of DR on reliability. [9] included line loss due to the integration of renewable energy but omitted the effect on reliability. Although [12] considered integrating renewable energy, with GA aid, the network reliability was missed.

It is observed that the influence of DR has been measured in terms of network performance, economic effects and GHG emission reduction. However, the impact of DR on the network performance variables has not been assessed, based on a combination of technical loss reduction, reliability, greenhouse emission and benefits of utility provider and consumer on grid-connected DG network. DR performance depends on various parameters such as cost, technical losses, GHG emission, and network reliability. Assessing DR impact should, therefore, be based on a combination of the variables mentioned above. This papers main target is to determine the effects of DR in the presence of integrated renewable energy based on the combination of cost, line losses, reliability, and GHG emission.

The contribution in this paper includes

- A new approach to identifying the optimal location of DG
- Determination of reduction in line loss due to the introduction DG and implementation of a DR program into a distribution system
- The introduction of a new function that combines the operation cost of electricity generation, GHG emission, technical loss, reliability and benefits of DR

The rest of the paper is presented in sections 2 to 6. Section 2 offers the mathematical modelling of the approach to the siting of DG and the cost associated with demand response on grid-connected DG. Section 3 explains the numerical stimulations, while section 4 presents the results. Section 5 and section 6 captures the discussion and the conclusion, respectively.

## 2. Mathematical modelling

### 2.1 Optimal location of DG

The optimal location of DG in a distribution network is determined based on the combination of voltage stability index (VSI) and power loss reduction index (PLRI). The VSI is based on the expression in Eq (1) [15] while the PLRI of the network is determined using Eq (2).

$$VSI=\frac{4(V_{o}V_{L}-V_{L}^{2})}{V_{o}^{2}}$$ (1)

where V_O_ and V_L_ represent the no-load and load voltage, respectively.

Power Loss Reduction index, (PLRI) = $\frac{P_{loss}-P_{loss,DG}}{P_{loss}}x\;100\%$

$$PLRI=\left(1-\frac{P_{loss,DG}}{P_{loss}}\right)x\;100\%$$ (2)

### 2.2 Grid-connected Distributed Generation (DG) operating cost

The network operating cost of a grid-connected microgrid includes transferring power from the national grid and the cost of running the microgrid. The operating cost of the DG is driven by the type of energy source employed. The generation sources assumed in this study are a diesel generator, wind generators and solar PV. Therefore, the operating cost of the DG is mainly the cost of fuel for the diesel generator. Other components that influence generation’s operating cost include the penalty to be paid for greenhouse gas (GHG) emission, cost of power loss during transmission, and cost of expected energy not supplied (CENS).

The operating cost of grid-connected DG (O_C_) is determined with the help of the equation expressed (3).

$$O_{c}=C_{i}(F_{t})+C_{r}({Pr}_{t})+\lambda_{s}(P_{loss,t}^{DG})+GEC_{t}+CENS_{t}$$ (3)

where:

- F_t_ is the fuel consumption at a time (t)
- Pr_t_ is the power transferred from the grid at any time (t)
- C_i_ is the unit cost of fuel
- C_r_ is the unit cost of energy transferred from an external grid
- *λ*_*s*_ is the selling price of energy
- $P_{loss,t}^{DG}$ is the distribution network power loss at a time (t)
- *GEC*_*t*_ is the penalty for greenhouse emission at a time (t)
- *CENS*_*t*_ is the cost of energy not supplied at a time (t)

Distribution companies, like any other industry, desire to minimize production costs. The minimum operation cost therefore, is obtained by minimizing function (f_1_) as indicated in Eq (4) subject to the power generating limits displayed in Eqs (5) to (8).

$$f_{1}=min\sum_{t=1}^{T}C_{i}(F_{t})+\sum_{t=1}^{T}C_{r}({Pr}_{t})+\lambda_{s}(P_{loss,t}^{DG})+GEC_{t}+CENS_{t}$$ (4)

$${Pr}_{min}\leq {Pr}_{t}\leq {Pr}_{max}$$ (5)

$${Pi}_{min}\leq {Pr}_{i,t}\leq {Pi}_{max}$$ (6)

$$0\leq {Pw}_{t}\leq W_{t}$$ (7)

$$0\leq {Ps}_{t}\leq S_{t}$$ (8)

where:

- Pr_rmin_ is minimum power that can be transferred from an external grid
- Pr_rmax_ is the maximum power that can be transferred from an external grid
- P_imin_ is minimum power that can be generated from a diesel generator
- P_imax_ is the maximum power that can be generated from a diesel generator
- W_t_ maximum power that can be generated from wind generator at time t’
- S_t_ maximum power that can be generated from solar at time ‘t’

### 2.3 Cost-benefit associated with Demand Response (DR)

#### 2.3.1 Cost-benefit of DR to consumers

The Consumers considered on the program can be categorized based on the willingness to curb power. The maximum capacity to be reduced is a measure of the readiness to reduce energy [2, 1] and ranges between ‘0’ and ‘1’[16]. The cost incurred by a consumer due to ‘x’kW power curtailed, is determined using Eq (9) [17].

$$c(\theta ,x)=K_{1}x^{2}+K_{2}x-K_{2}x\theta$$ (9)

where:

- c(θ,x) is the cost incurred
- K_1_ and K_2_ represent cost coefficients
- θ is the category of consumer
- x is power curtailed in ‘kW’

The benefit derived by consumers on the program is expected to be according to Eq (10).

$$b_{j}=y_{j}-\left(K_{1}x^{2}+K_{2}x-K_{2}x\theta \right);\;\mathrm{for}\;j=1,2,\ldots ,J$$ (10)

- b_j_ is a benefit derived by a consumer
- y_j_ is an incentive received by a consumer

#### 2.3.2 Cost-benefit of DR to a utility provider

The benefit of the program to the distribution companies is based on the cost of not supplying power to a location (λ) and the amount paid as compensation (y) under DR. The utility benefit derived from each consumer on the program is according to Eq (11)

$$b_{u}(\theta ,\lambda )=(\lambda x-y)$$ (11)

where:

- b_u_(θ,λ) is the benefit derived due to each type of consumer on the program

The total utility benefit for ‘j’ consumers on the program, at a time,(t) is based on the function in Eq (12).

$$b_{u}=[\sum_{j=1}^{J}\left(\lambda_{j,t}x_{j,t}-y_{j,t}\right)]$$ (12)

The utility companies can derive maximum benefit if the incentive to be paid is minimum, hence the difference between the cost of power not supplied and compensation paid is maximum as indicated by the function (f_2_) in Eq (13).

$$f_{2}=max_{x,y}[\sum_{t=1}^{T}\sum_{j=1}^{J}\left(\lambda_{j,t}x_{j,t}-y_{j,t}\right)]$$ (13)

Subject to the following constraints

- The consumer compensation must not be less than the cost incurred by the consumer due to power curtailed. The relationship between energy curtailed and compensation paid to each consumer is according to the constraint stated in Eqs (14) and (15). Eq (15) ensures that the incentive received by a consumer, more willing to reduce power consumption, is higher than the compensation paid to the consumer with less willingness.

$$\sum_{t}^{T}\left[y_{j,t}-\left(K_{1j}x_{j,t}{}^{2}+K_{2j}x_{j,t}-K_{2j}x_{j,t}\theta_{j}\right)\right]\geq 0\;\mathrm{for}\;j=1,2,\ldots ,J$$ (14)

$$\sum_{t}^{T}\left[y_{j,t}-\left(K_{1j}x_{j,t}{}^{2}+K_{2j}x_{j,t}-K_{2j}x_{j,t}\theta_{j}\right)\right]\geq \sum_{t}^{T}[y_{j-1,t}-(K_{1j-1}x_{j,t}{}^{2}+K_{2j-1}x_{j-1,t}+K_{2j-1}x_{j-1,t}\theta_{j-1})]\;\mathrm{for}\;j=2,3,\ldots ,J$$ (15)

- The total power curtailed at any given location must not exceed the consumers agreed maximum limit (CMj). The maximum power curtailed for each consumer is regulated with the constraint stated in Eq (16).

$$\sum_{t=1}^{T}x_{j,t}\leq CM_{j}$$ (16)

- The utility company’s total compensation is within budget (UB), as stated by Eq (17).

$$\sum_{t=1}^{T}\sum_{j=1}^{T}\lambda_{j,i}\leq UB$$ (17)

### 2.4 Demand response on grid-connected DG network

**Fig 1:**
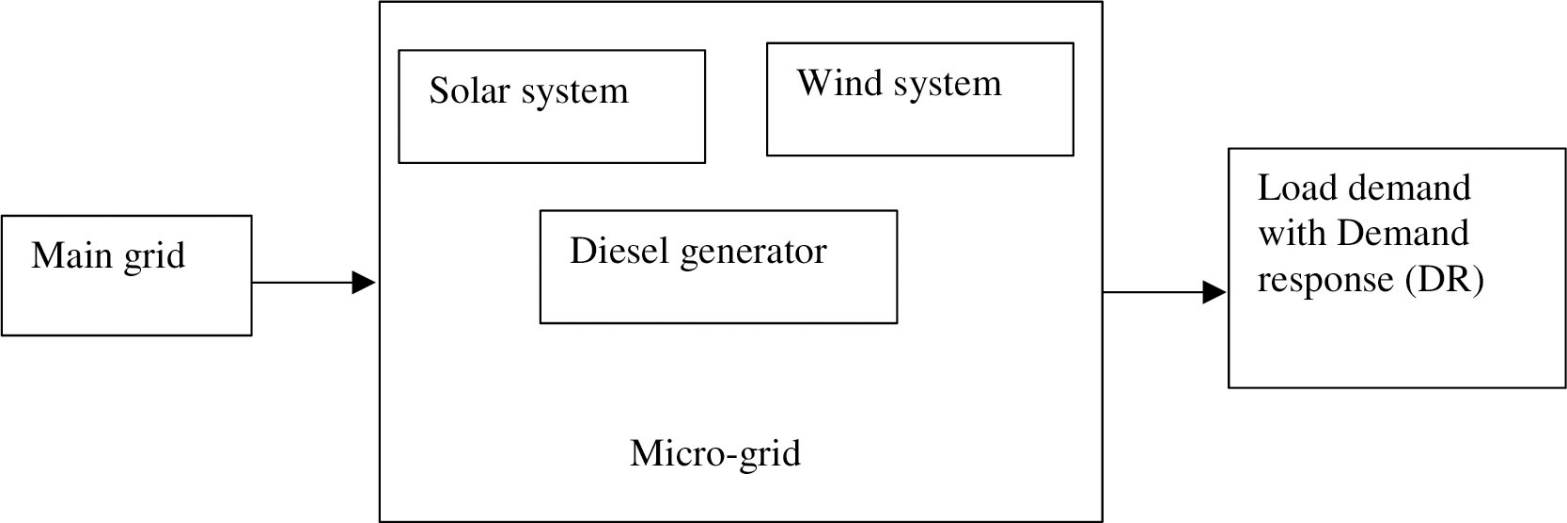
Grid-connected microgrid with demand response setup.

Demand response as applied to grid-connected DG is as illustrated by Fig 1. Utility providers derive the maximum benefit if the operating cost and the compensation paid to consumers on the demand response program are minima. Therefore, the utility maximum benefit function based on the integration of demand response is expressed as Eq (18). This function is an extention from [8]. The difference is that the cost of generation includes line losses, GHG emission and cost of energy not supplied.

$$f_{3}=min\;w_{1}[\sum_{t=1}^{T}C_{i}(F_{t})+\sum_{t=1}^{T}C_{r}({Pr}_{t}){+\lambda }_{s}(P_{loss,t}^{DG})+GEC_{t}+CENS_{t}]-w_{2}[\sum_{t=1}^{T}\sum_{j=1}^{J}\left(\lambda_{j,t}x_{j,t}-y_{j,t}\right)]$$ (18)

where the weights sum up to 1 as indicated in Eq (19).

$$w_{1}+w_{2}=1$$ (19)

Where

*w*_1_ is the weight attached to cost associated with power generation
*w*_2_ is the weight attached to financial benefits derived

The ‘f_3_’function is subject to the constraints stated as Eqs (5) to (8) as well as Eqs (14) to (17). Additionally, the load served is regulated according to the total power from available energy sources. The load regulation is by the implementation of the constraint expressed in Eq (20).

$$P_{i,t}+{Pw}_{t}+{Ps}_{t}+{Pr}_{t}=D_{t}-\sum_{j=1}^{J}X_{j,t}$$ (20)

where:

- D_t_ represents the actual load demand at a time.
- w_1_ and w_2_ are the weights attached to the combined objectives
- *X*_*j*,*t*_ represents the power curtailed by each consumer at a time, t.

### 2.5 Environmental impact of demand response

The environmental influence of DR is assessed base on the level of GHG introduced into the atmosphere under the implementation of the DR program. The gases of concentration in this study are carbon dioxide (CO_2_), sulfur dioxide (SO_2_) and Nitrogen oxide (NO_x_). The penalty to be paid by a utility provider is to be estimated using Eq (21)

$$GEC=\sum_{i=1}^{n}\sum_{g=1}^{n}PF_{g}P_{i}(CO_{2Gi(t)}+SO_{2Gi(t)}+NO_{xGi(t)})$$ (21)

where:

PF_j_ is the cost incurred for the emission of the various types of gases‘g’ in GH¢/kg as indicated in Table 1.

P_i_ is the output power from a diesel generator

‘n’ refers to the total number of generators.

CO_2Gi(t)_, SO_2Gi(t)_ and NO_xGi(t)_ represent the various level of the differentgases emitted based on the assumed emission factors indicated in Table 1.

**Table 1:** Emission cost and emission factor of diesel generator [18, 19].

Type of gas	Emission cost (GHȼ/kg)	Emission factor (kg/kWh)
CO_2_	0.09214	0.6569395
SO_2_	9.756	0.0003595
NO_x_	5.90238	0.0066911

### 2.6 Reliability of distribution network

Reliability is an essential indicator used for measuring the performance of a distribution network. One of the reliability measurements is the availability of electricity supply to end-users. Implementation of demand response programs can contribute to reducing the peak load. This action can potentially influence the availability of electricity to the end-users. The effect of implementing demand response (DR) program on reliability is assessed based on indices, such as expected energy not supplied, loss of load expectation and loss of load probability.

- Expected energy not supplied (EENS), refers to the quantity of energy not supplied in a year [20]. The value is estimated using Eq (22) [21]

$$EENS=\sum_{k}^{K}P_{k}\times E_{k}$$ (22)

$$CENS=\lambda_{E}\;EENS$$ (23)

- Loss of load expected (LOLE) represents the number of hours when the demand load exceeds the supply. It is estimated with the help of Eq (24) [22].

$$LOLE=\sum_{k}^{n}P_{k}\times t_{k}$$ (24)

- Loss of load probability (LOLP) is the probability that demand power will exceed the supply within a defined period. It is calculated using Eq (25).

$$LOLP=\sum_{k}P_{k}\times t_{j}$$ (25)

Where:

P_k_ is the outage probability

E_k_ is the energy curtailed

λ_E_ is the profit per kWh

t_k_ is the time when a load is lost.

t_j_ is a percentage of the time when the load exceeds supply.

## 3. Simulations

The extension of electricity to all parts of a country is essential to promoting its economic and social development. The extension of electricity in Ghana will create an enabling environment to enable the government to set up factories in all the country’s districts as planned. Adequate electricity provision to end uses sometimes requires implementing a load management technique, including demand response (DR) program. The influence of DG is determined based on a function combining operation cost, technical loss, GHG emission reliability and financial benefits of both utility and the consumer.

The Ghanaian distribution network under consideration currently has no DG connected and incurs active power losses equivalent to about 13% of total end loads. When the demand loads exceed available energy for distribution, load shedding is adopted to manage the situation. S1 Appendix presents the structure of the 167 bus distribution network under consideration. S2 Appendix shows the line losses of the network under consideration. The appendix also presents the influence of DG (location selected according to Eqs (1) and (2)) influence on the line losses of the network. Currently, individual electricity from micro-generators is not allowed to feedback into the national grid. Hence it is assumed that the power can only be transferred in only one direction from the national grid to a microgrid.

This study examines two electricity demand cases. The first case is a low electricity demand, assumed to be for domestic purposes. The second case is a typical small scale rural industrial load demand on a Ghanaian distribution network. The sub-cases considered under each case is as shown in Table 2.

**Table 2:** Maximum specifications of cases considered in this study.

	Source of energy			Number of consumers on DR program	Utility budget limit [GhȻh
Case 1					
A	Diesel				
B	Diesel			3	4,000
C	Diesel	Solar	Wind	3	4,000
Case 2					
A	Diesel				
B	Diesel			7	400,000
C	Diesel	Solar	Wind	7	400,000

The technical loss experienced in the distribution network depends on the location of DG in the distribution network and the load supplied. The optimal location of DG in the distribution network was based on Eqs (1) and (2). Due to the assumed DG penetration level of 20%, the power loss was determined by simulation with Power System Analysis Toolbox (PSAT) on a MATLAB platform. The assessments of the cost of GHG, reliability, energy saved and incentive paid were, on the other hand, determined with the help of Advanced Interactive Multidimensional Modelling System (AIMMS). Both simulating software were installed on a computer with Intel(R), Core (TM), i5-4310U and installed memory (RAM) of 8GB.

The simulation in AIMMS was based on the following assumptions;

- The fuel cost for running the diesel generator is assumed (based on diesel price at the Goil filling station as at 31/12/2019) to be GH¢5.36 per litre.
- The distribution network assets can support up to 75% of peak load.
- The energy supply is to be transferred from an external grid at an assumed cost of GH¢0.29 per kWh and sold at GH¢0.65 per kWh [23].
- The utility companies have information on the maximum energy curtailed per day (CMj) per consumer. The value of CMj, per consumer, forms the basis for determining the various consumer willingness. The utility is also assumed to know the cost function coefficients (K_1_ and K_2_) of each consumer included in the program.
- Three (3) domestic consumers agree to be on the demand response program, while seven (7) rural industrial consumers are on the demand response program.
- The utility budget is limited to GHȻ4,000 for compensation to domestic consumers and GH¢400,000 for the assumed rural industrial consumers.

### 3.1 Case one

This case considers a low demand profile network with electricity demand ranging from 195kW to 285kW. The cost coefficients K_1_ and K_2_ are as indicated in Table 3. The table also presents the maximum energy that can be curtailed to a location, CMj. The hourly load demand, the network value, assumed full wind power and solar power are shown in Table 4.

**Table 3:** Cost coefficients, consumer classification and limit of curtail power to consumers—case 1 [8].

Consumer, j	*K*_1*j*_	*K*_2*j*_	*θ*_*j*_	*CM*_*j*_(*kWh*)
1	1.079	1.32	0	30
2	1.378	1.63	0.45	35
3	1.847	1.64	0.9	40

**Table 4:** Hourly load demand, cost of not supplying power to a consumer, wind power and solar power–case 1.

Time (hour)	Initial Load Demand (kW)	Cost of power not supplied, *λ*_*j*,*t*_ (Gh¢)	Wind power, W_t_, (kW)	Solar power, S_t_, (kW)
1	229.71	8.51	7.08	
2	226.29	7.59	5.02	
3	223.29	11.92	12.16	
4	220.71	20.38	11.63	
5	224.14	24.39	10.37	
6	235.29	25.47	5.6	
7	221.14	27.32	10.08	40.6323
8	200.14	29	16.27	132.285
9	195.86	36.31	25.87	223.312
10	199.29	33.39	30.49	306.226
11	202.71	34.58	39	363.932
12	205.29	36.96	39.28	386.652
13	207.86	39.57	49.74	397.135
14	199.71	42.28	61.24	366.213
15	215.14	46.07	52.13	315.03
16	203.14	38.48	50.24	239.451
17	218.14	36.86	43.95	142.77
18	225.43	34.15	28	51.8462
19	258.86	31.44	31.22	
20	285	22.76	25.89	
21	279.86	20.6	15.94	
22	268.29	16.31	10.59	
23	253.29	13.71	7.63	
24	240	7.7	7.88	

### 3.2 Case two

This case considers the average hourly load demand of the network in Table 5, with an associated hourly value of interruption cost. The table also shows the maximum hourly solar and wind power assumed can be generated. Table 6 shows the cost coefficients and the limits regarding energy curtailed per consumer.

**Table 5:** Hourly load demand, cost of not supplying power to a consumer, wind power and solar power–case 2.

Time (hour)	Initial Load Demand (kW)	Cost of power not supplied, *λ*_*j*,*t*_(Gh¢)	Wind power, W_t_, (kW)	Solar power, S_t_, (kW)
1	2297.14	25.53	28.33	
2	2262.86	22.77	20.10	
3	2232.86	35.76	48.64	
4	2207.14	61.14	46.52	
5	2241.43	73.17	41.50	
6	2352.86	76.41	22.42	
7	2211.43	81.96	40.32	121.90
8	2254.29	87.00	65.06	396.85
9	2588.57	108.93	103.49	669.93
10	2850	100.17	121.94	918.68
11	2798.57	103.74	156.02	1091.80
12	2682.86	110.88	157.13	1159.95
13	2532.86	118.71	198.94	1191.40
14	2400	126.84	244.95	1098.64
15	2151.43	138.21	208.53	945.09
16	2031.43	115.44	200.98	718.35
17	2181.43	110.58	175.79	428.31
18	2001.43	102.45	112.02	155.54
19	1958.57	94.32	124.87	
20	1992.86	68.28	103.55	
21	2027.14	61.8	63.77	
22	2052.86	48.93	42.35	
23	2078.57	41.13	30.50	
24	1997.14	23.10	31.51	

**Table 6:** Cost coefficients, consumer classification and limit of curtail power to consumers—case 2 [8].

Consumer, j	*K*_1*j*_	*K*_2*j*_	*θ*_*j*_	*CM*_*j*_(*kWh*)
1	1.847	11.64		580
2	1.378	11.63	0.14	630
3	1.079	11.32	0.26	710
4	0.9124	11.5	0.37	790
5	0.8794	11.21	0.55	840
6	1.378	11.63	0.84	930
7	1.5231	11.5	1	1000

### 3.3 Sensitivity analysis

The base case simulation is by attaching equal importance to the cost of providing electricity to the end load and the benefit derived from the implementation of DR. The influence of varying the significance attached to the cost of providing electricity and the benefit derive from DR was determined by ranging the weight (w) from 0 to 1. Where w = 0 represents minimum importance to the cost of electricity provision and maximum attention to DR benefits. Alternatively, w = 1 means that utmost attention is attached to the cost of electricity and minimum importance attached to the DR benefits. The ideal value to be attached to the weights will be the values corresponding to the point where Eq (18) is minimum.

The influence of the maximum power that can be curtailed by each consumer was also studied. The evaluation of energy shortened impact was by varying the maximum energy allowed to be shrunk, for each consumer, by ±5% and ±10%. The variable CM used for this analysis is, as shown in Tables 7 and 8.

The sensitivity investigation was carried out under case 1c and case 2c.

**Table 7:** CM variable for sensitivity analysis–case 1.

	CM 1	CM 2	CM 3	CM 4	CM 5
C1	25.65	28.5	**30**	31.5	33
C2	29.925	33.25	**35**	36.75	38.5
C3	34.2	38	**40**	42	44

**Table 8:** Variable CM used under sensitivity–case 2.

	CM 1	CM 2	CM 3	CM 4	CM 5
C1	495.9	551	**580**	609	638
C2	538.65	598.5	**630**	661.5	693
C3	607.05	674.5	**710**	745.5	781
C4	675.45	750.5	**790**	829.5	869
C5	718.2	798	**840**	882	924
C6	795.15	883.5	**930**	976.5	1023
C7	855	950	**1000**	1050	1100

## 4. Results

This work investigates the effect of DR in the presence of renewable energy on the management of technical losses, supply to load demand balance, and reliability of power supply without overlooking the emission of greenhouse gas (GHG). The optimal location for installing DG in the Ghanaian 167-bus distribution network is bus 112. The Eq (18) with equal weight (w = 0.5) attached to the objectives as in Eq (19) is the bases for the other simulation results. The following sections discuss the effects of various situations.

### 4.1 Power supply

**Fig 2:**
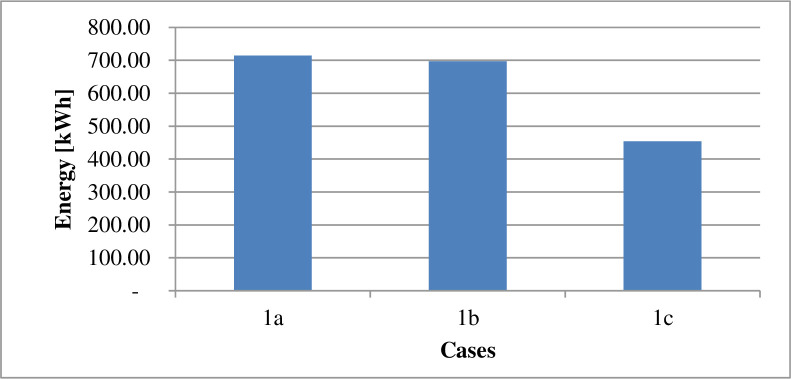
Energy losses per day–case 1.

**Fig 3:**
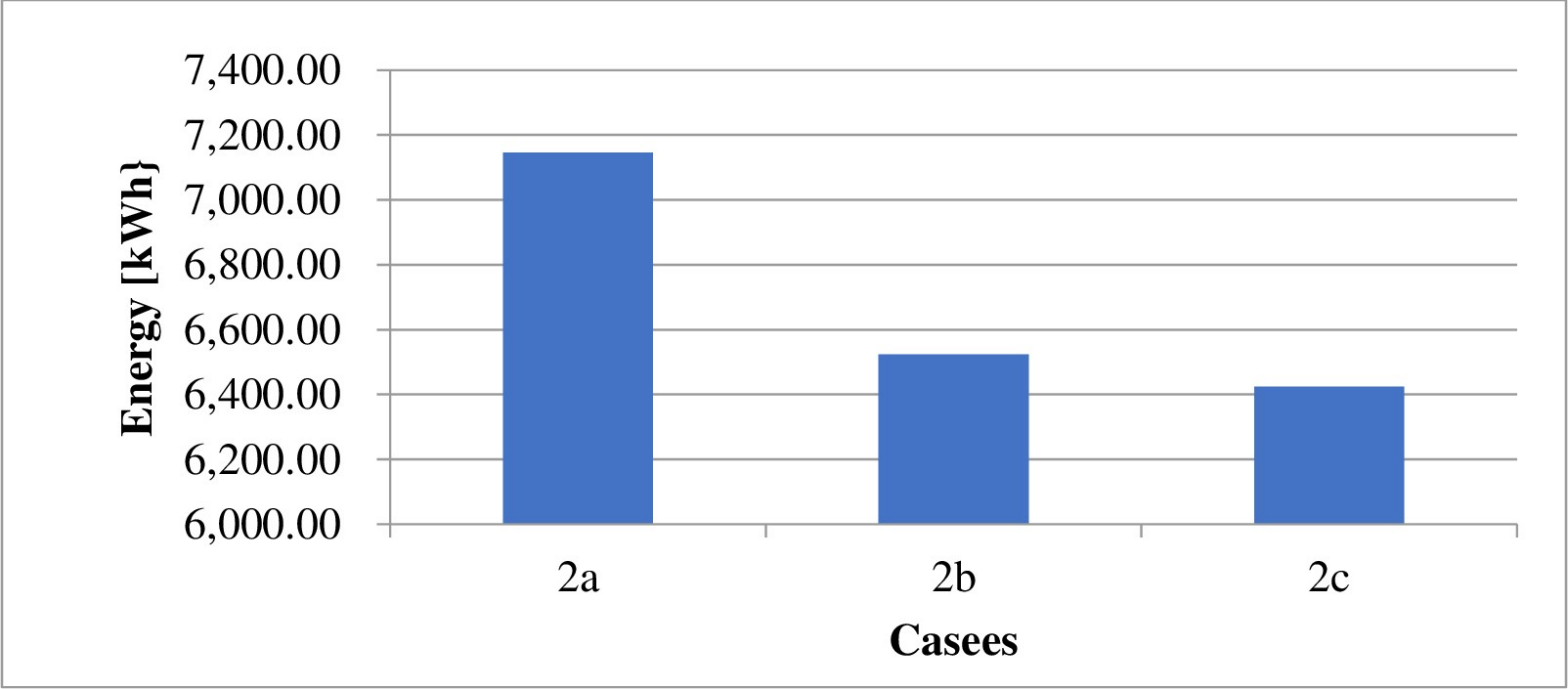
Energy losses per day–case 2.

**Fig 4:**
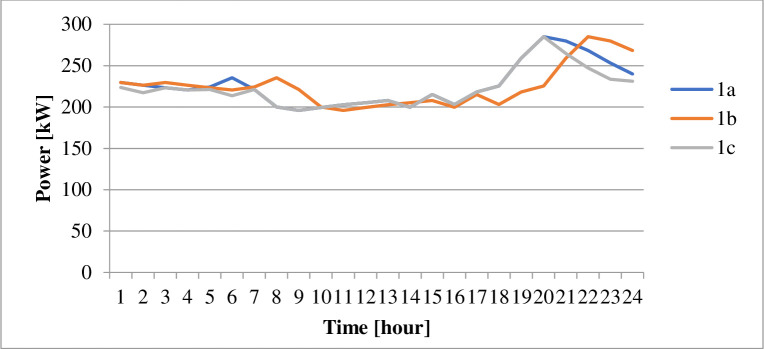
Load profiles within 24 hours–case 1.

**Fig 5:**
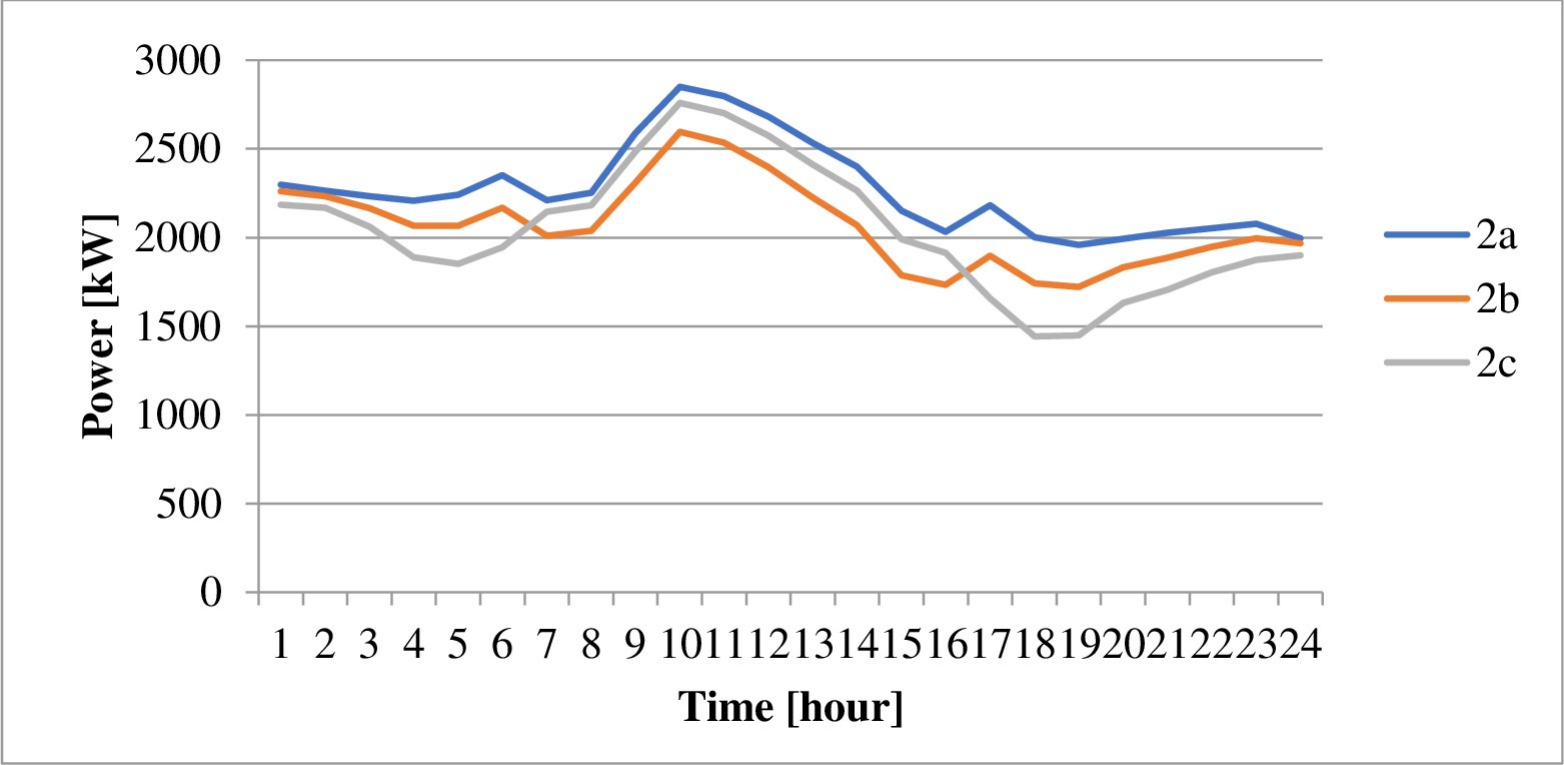
Load profile within 24 hours–case2.

**Fig 6:**
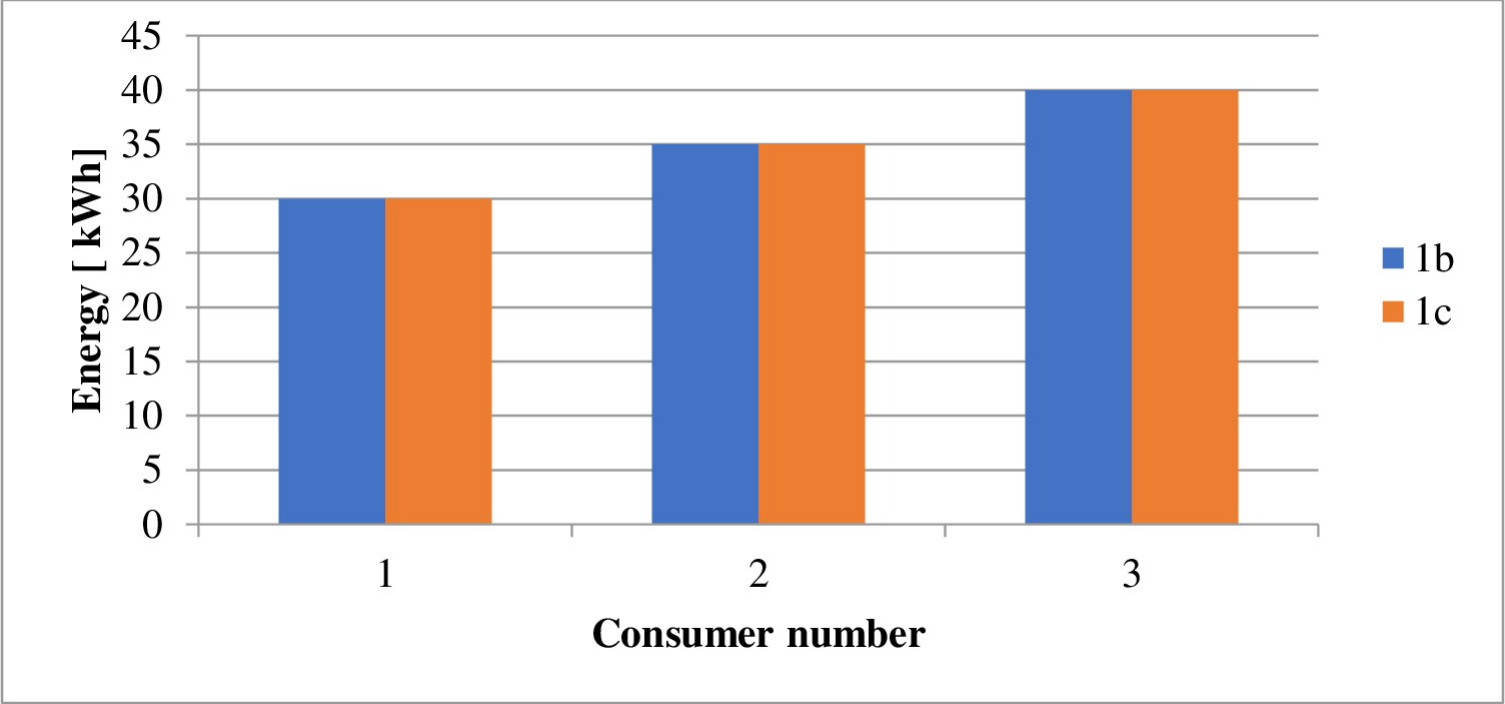
Power curtailed by consumers per day–case 1.

**Fig 7:**
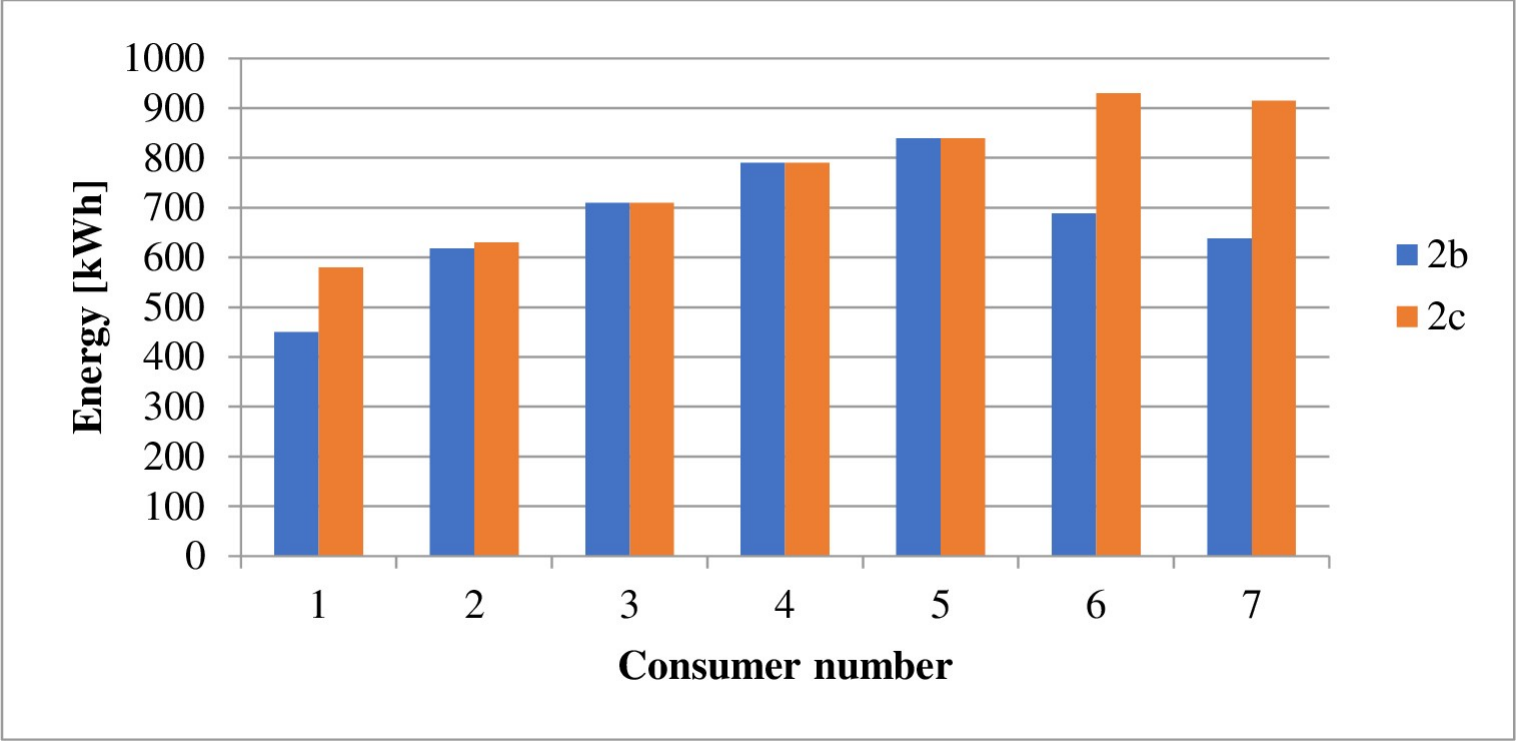
Power curtailed by consumers per day–case 2.

The energy derived from the different sources under the various cases is as displayed in Table 9. The power losses associated with each scenario is as indicated by Figs 2 and 3. Figs 4 and 5 present the load profile under each case. Additionally, Figs 6 and 7 illustrate the energy saved per day with the implementation of DR and incorporating renewable energy under case1 and case 2.

**Table 9:** Energy supply under various cases.

	1a	1b	1c	2a	2b	2c
Total energy at load point [kWh]	5,413.31	5,308.31	53,19.341	54,133.09	49,649.21	48,895.71
Diesel energy [kWh]	1,087.716	1,087.716	416.9567	10,877.15	10,877.15	3,943.439
Grid energy [kWh]	4,325.594	4220.594	4,252.625	43,255.94	38,772.06	39,097.53
Solar energy [kWh]	0	0	378.05	0	0	4,218.134
Wind energy [kWh]	0	0	271.7093	0	0	1,636.601

### 4.2 Finance

**Fig 8:**
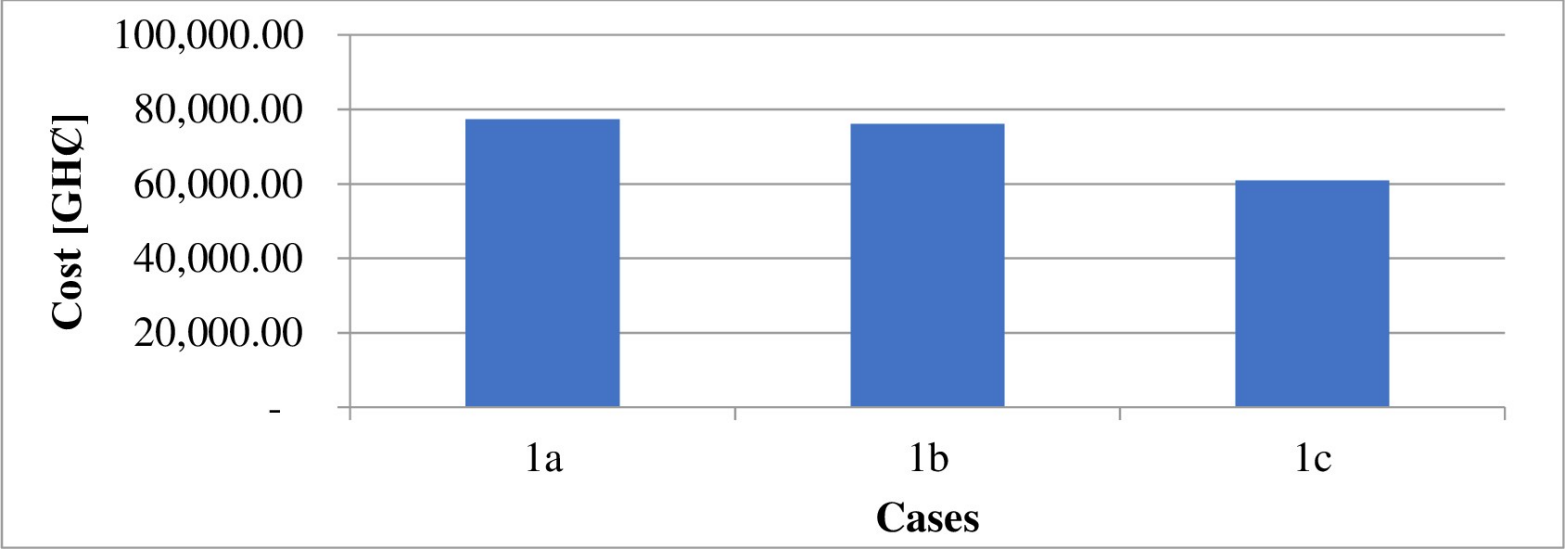
Cost of greenhouse emission–case 1.

**Fig 9:**
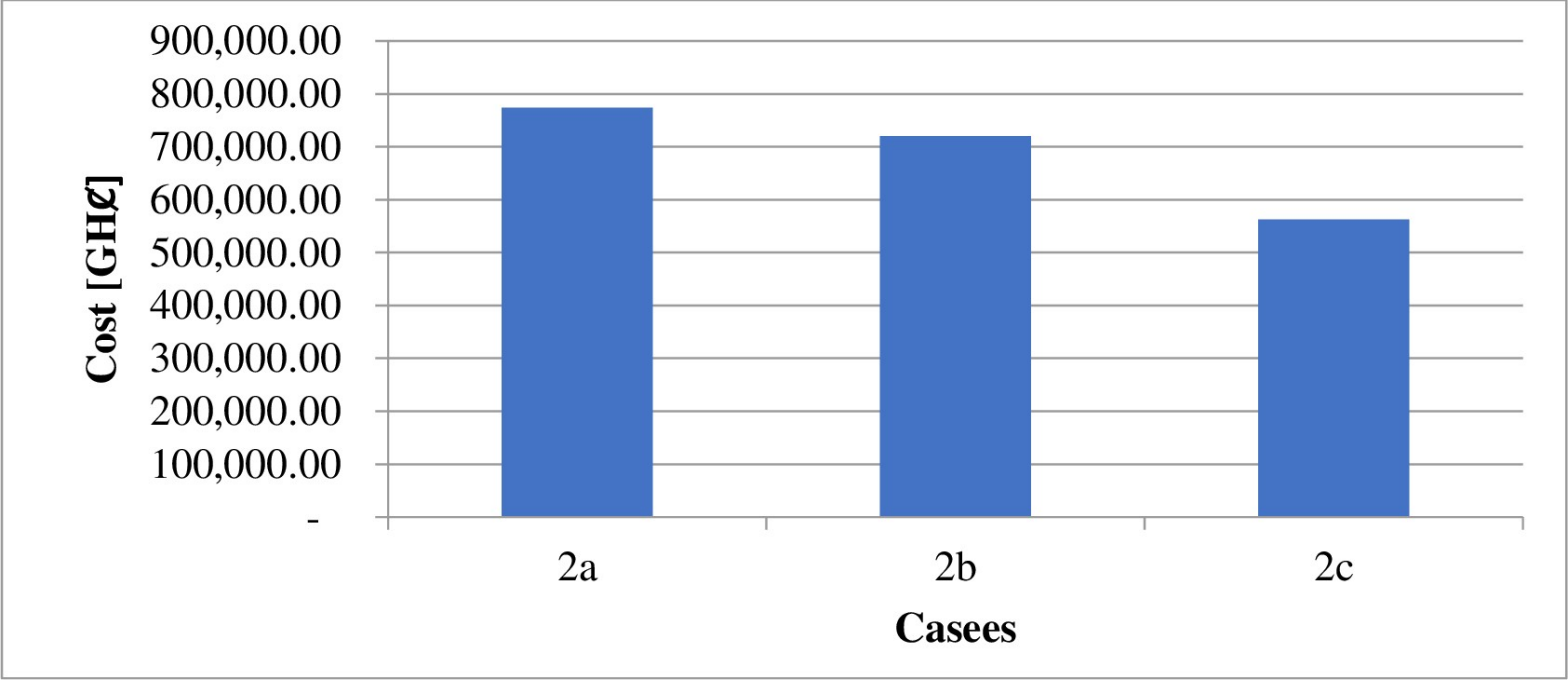
Cost of greenhouse gases emission–case 2.

**Fig 10:**
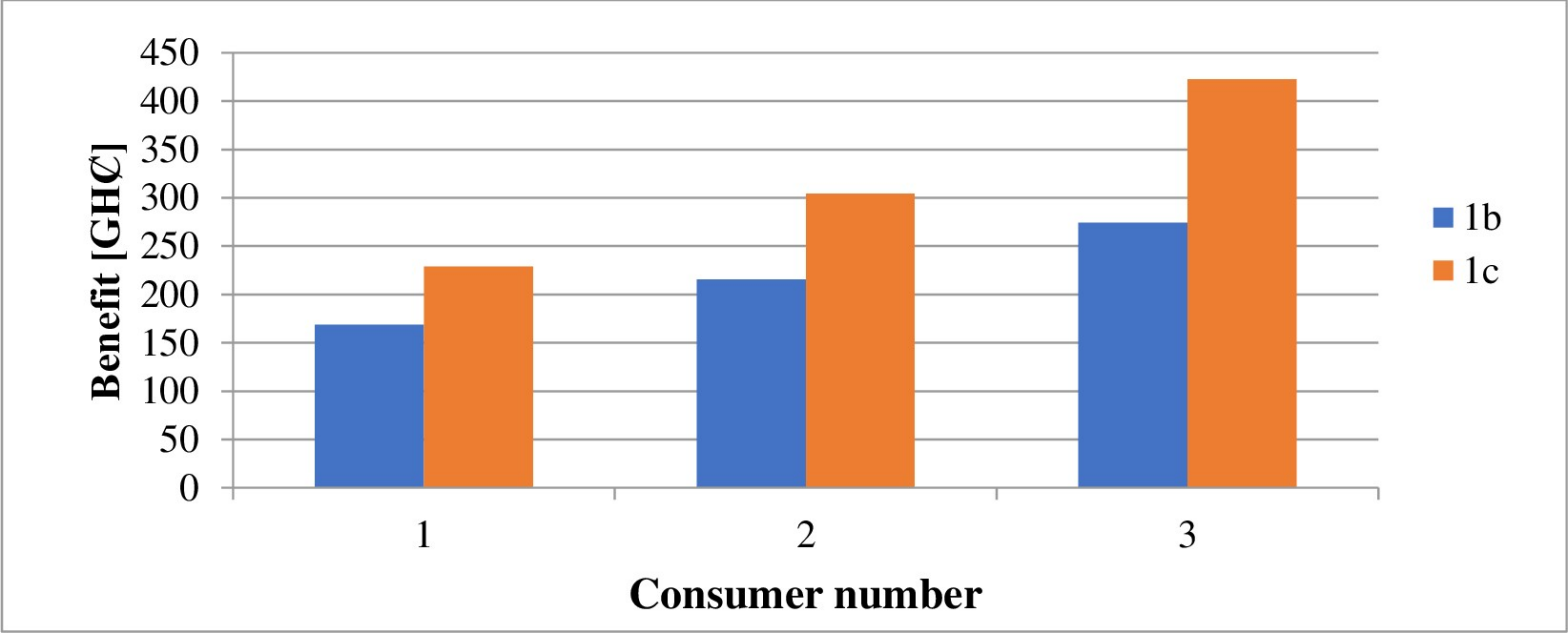
Incentives paid consumers per day–case 1.

**Fig 11:**
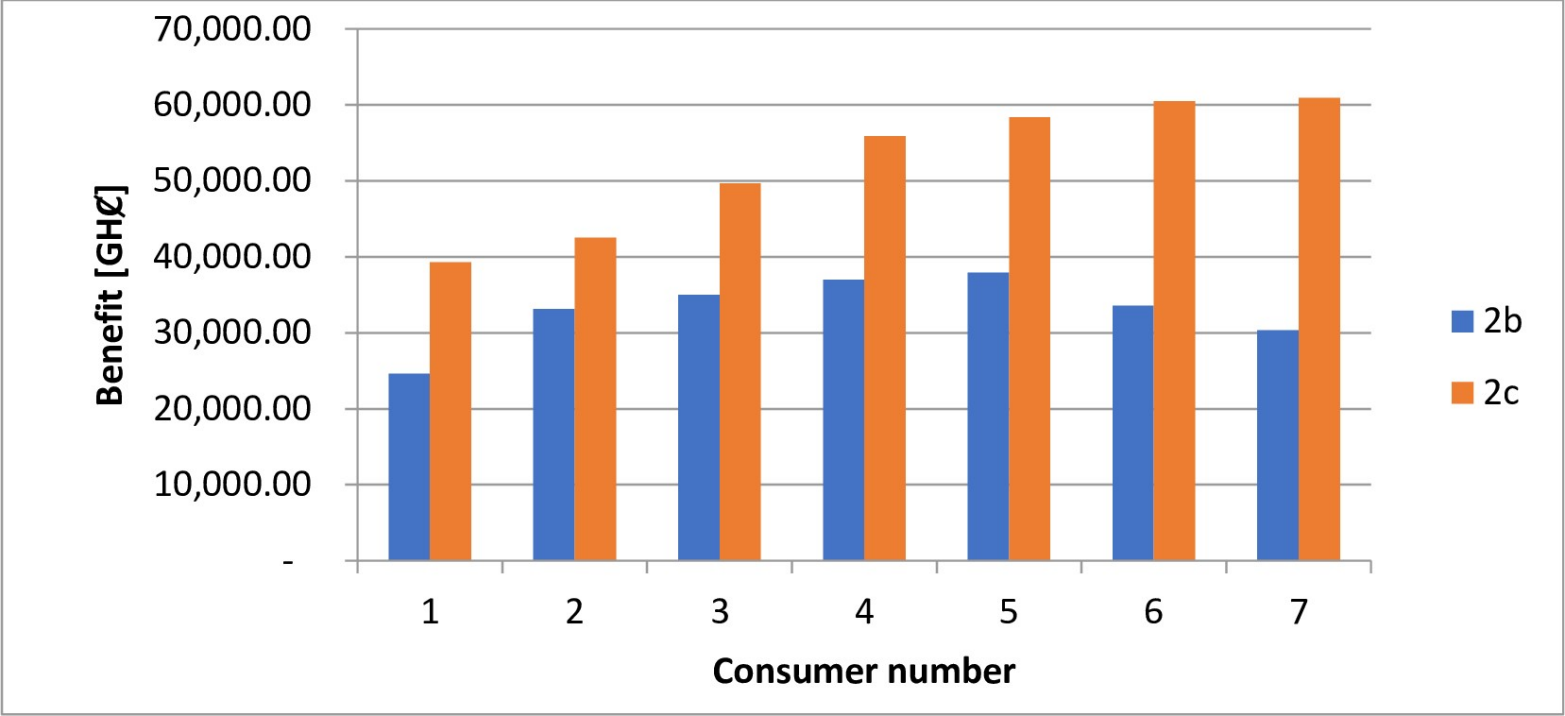
Incentives paid to consumers per day–case 2.

The estimated financial components associated with the daily generation and distribution of electricity in each case are in Table 10. Figs 8 and 9 illustrate the costs of GHG due to electricity generation under different circumstances. The incentives paid to each consumer participating in the DR program under case 1 and case 2 are shown in Figs 10 and 11.

**Table 10:** Finances associated with each case.

	1a	1b	1c	2a	2b	2c
Operation cost [GH¢]	1,946,794	1,945,486.08	721,774.58	78,306,159.47	78,250,693.65	21,672,397.11
Diesel cost [GH¢]	1,867,658	1,867,657.89	659,111.07	77,514,795.14	77,514,795.14	21,093,732.73
Transfer cost [GH¢]	1,254.422	1,223.97	1,233.26	12,544.22	11,243.90	11,338.28
Cost of loss [GH¢]	464.5091	453.38	698.96	4,645.09	4,030.99	4,176.18
Utility benefit	0	3,427.67	789.86	-	230,801.02	75,449.07

### 4.3 Reliability

Table 11 presents the influence of DR on the reliability of the distribution networks considered in this study.

**Table 11:** Influence of DR on reliability.

	1a	1b	1c	2a	2b	2c
EENS	25.27	25.27	14.24	252.64	-	95.16
CENS	9.10	9.10	5.13	90.95	-	34.26
LOLE	3.00	3.00	1.00	3.00	-	2.00
LOLP	0.00	0.00		0.00	-	0.00

### 4.4 Sensitivity

The Tables 12 and 13 presents the influence of varying the weight (w) attached to the main objectives, on the grid-connected DG network. Tables 14 and 15 also show the effects of the DR program’s consumers, changing the limits of power that can be curtailed under case 1 and case 2. The base case results of the sensitivity analysis are bold.

**Table 12:** Influence of ’w’ on distribution network–case 1.

	w = 0	w = 0.1	w = 0.2	w = 0.3	w = 0.4	w = 0.5	w = 0.6	w = 0.7	w = 0.8	w = 0.9	w = 1.0
Diesel cost [GHȻ]	703,138.71	661,754.37	660,282.00	659,564.31	659,264.19	**659,111.07**	659,046.97	659,020.21	659,009.83	659,003.59	659,001.46
Grid energy transfer cost [GHȻ]	1,224.06	1,235.67	1,234.88	1,233.94	1,233.38	**1,233.26**	1,233.26	1,233.26	1,233.21	1,232.95	1,232.70
Cost of loss [GHȻ]	453.39	455.04	454.80	454.52	454.36	**454.32**	454.32	454.32	454.31	454.23	454.16
GEC [GHȻ]	61,176.18	61,076.26	61,043.44	61,004.06	60,980.99	**60,975.93**	60,975.93	60,975.93	60,973.65	60,963.15	60,952.77
Diesel generator energy [kWh]	437.83	416.96	416.96	416.96	416.96	**416.96**	416.96	416.96	416.96	416.96	416.96
External grid energy [kWh]	4,220.90	4,260.94	4,258.22	4,254.96	4,253.04	**4,252.62**	4,252.62	4,252.62	4,252.44	4,251.57	4,250.70
Solar energy [kWh]	378.93	380.04	380.04	380.04	380.04	**380.04**	380.04	380.04	380.04	380.04	380.04
Wind energy [kWh]	270.69	269.72	269.72	269.72	269.72	**269.72**	269.72	269.72	269.72	269.72	269.72
CENS [GHȻ]	9.08	2.13	3.11	4.29	4.98	**5.13**	5.13	5.13	5.19	5.51	5.82
Energy curtailed [kWh]	105.00	105.00	105.00	105.00	105.00	**105.00**	105.00	105.00	105.00	105.00	105.00
Incentives paid [GHȻ]	663.67	721.22	746.06	825.82	897.73	**956.27**	994.63	1,017.21	1,029.84	1,046.52	1,110.53

**Table 13:** Influence of ’w’ on distribution network–case 2.

	w = 0	w = 0.1	w = 0.2	w = 0.3	w = 0.4	w = 0.5	w = 0.6	w = 0.7	w = 0.8	w = 0.9	w = 1.0
Diesel cost [GHȻ]	24,941,944.50	21,337,561.23	21,135,364.18	21,110,540.82	21,097,210.28	**21,093,732.73**	21,093,732.73	24,941,944.50	21,337,561.23	21,135,364.18	21,110,540.82
Grid energy transfer cost [GHȻ]	11,457.23	11,355.44	11,343.44	11,339.98	11,338.69	**11,338.28**	11,338.15	11,457.23	11,355.44	11,343.44	11,339.98
Cost of loss [GHȻ]	4,241.33	4,181.59	4,177.82	4,176.70	4,176.31	**4,176.18**	4,176.13	4,241.33	4,181.59	4,177.82	4,176.70
GEC [GHȻ]	579,186.90	565,593.93	564,015.44	563,523.49	563,236.49	**563,149.91**	563,144.44	579,186.90	565,593.93	564,015.44	563,523.49
Diesel generator energy [kWh]	4,421.42	4,018.02	3,971.50	39,103.38	3,946.45	**3,943.44**	3,943.44	4,421.42	4,018.02	3,971.50	39,103.38
External grid energy [kWh]	39,507.69	39,156.70	39,115.31	3,956.50	39,098.92	**39,097.53**	39,097.08	39,507.69	39,156.70	39,115.31	3,956.50
Solar energy [kWh]	4,084.56	4,151.82	4,198.99	4,212.76	4,222.74	**4,225.66**	4,225.51	4,084.56	4,151.82	4,198.99	4,212.76
Wind energy [kWh]	1,644.76	1,632.46	1,629.08	1,629.08	1,629.08	**1,629.08**	1,629.08	1,644.76	1,632.46	1,629.08	1,629.08
CENS [GHȻ]	-	18.89	29.86	32.39	33.90	**34.26**	34.22	-	18.89	29.86	32.39
Energy curtailed [kWh]	4,727.30	5,374.25	5,387.90	5,394.05	5,394.39	**5,394.86**	5,395.57	4,727.30	5,374.25	5,387.90	5,394.05
Incentives paid [GHȻ]	230,904.29	321,919.55	350,060.29	358,370.00	364,924.53	**367,255.21**	367,333.01	230,904.29	321,919.55	350,060.29	358,370.00

**Table 14:** Influence of varying CM on a network–case 1.

	CM 1	CM 2	CM 3	CM 4	CM 5
Diesel cost [GHȻ]	659,111.07	661,319.52	**659,111.07**	656,907.68	654,711.82
Grid energy transfer cost [GHȻ]	1,236.79	1,234.48	**1,233.26**	1,232.04	1,230.83
Cost of loss [GHȻ]	454.32	454.77	**454.32**	453.88	453.43
GEC [GHȻ]	60,975.93	61,050.95	**60,975.93**	60,900.90	60,825.87
Diesel generator energy [kWh]	416.96	418.01	**416.96**	415.91	414.86
External grid energy [kWh]	4,252.62	4,256.82	**4,252.62**	4,248.42	4,244.22
Solar energy [kWh]	380.04	380.04	**380.04**	380.04	380.04
Wind energy [kWh]	269.72	269.72	**269.72**	269.72	269.72
CENS [GHȻ]	5.13	5.13	**5.13**	5.13	5.13
Energy curtailed [kWh]	105.00	99.75	**105.00**	110.25	115.50
Incentives paid [GHȻ]	956.27	883.16	**956.27**	1,034.15	1,113.20

**Table 15:** Influence of varying CM on a network–case 2.

	CM 1	CM 2	CM 3	CM 4	CM 5
Diesel cost [GHȻ]	21,246,146.07	21,110,985.57	**21,093,732.73**	21,093,732.73	21,093,732.73
Grid energy transfer cost [GHȻ]	11,485.63	11,385.18	**367,255.21**	11,293.51	11,257.69
Cost of loss [GHȻ]	4,231.70	4,193.82	**4,176.18**	4,159.17	4,145.49
GEC [GHȻ]	570,620.82	565,410.65	**563,149.91**	561,287.48	4,145.49
Diesel generator energy [kWh]	4,001.26	3,956.80	**3,943.44**	3,943.44	3,943.44
External grid energy [kWh]	39,605.62	39,259.23	**367,255.21**	38,943.15	38,819.62
Solar energy [kWh]	4,309.82	4,257.19	**3,943.44**	4,180.95	4,144.31
Wind energy [kWh]	1,628.98	1,628.98	**1,628.98**	1,628.98	1,628.98
CENS [GHȻ]	55.67	40.56	**34.26**	25.32	17.03
Energy curtailed [kWh]	4,685.40	5,170.86	**5,394.86**	5,618.86	5,802.06
Incentives paid [GHȻ]	308,920.04	351,641.05	**367,255.21**	378,310.07	386,730.60

## 5. Discussion

The base case is the 20% penetration of DG introduced into the distribution networks based on diesel generator electricity, as shown in Table 5 as 1a for case 1 and 2a for case 2. The discussion of results obtained under each option is in the following sections.

### 5.1 Case one

According to Table 9, the DR program implementation caused the energy transferred from an external grid to reduce by 105kWh per day. The DR program’s performance is responsible for the 2.43% reduction in the grid’s transmitted energy. The DR program’s presence also caused a decrease of GHȻ1,308.27 in the network operation cost, as suggested in Table 10. According to Fig 2, the DR program introduction (1b), caused a 2.4% reduction in the technical losses. The resultant load variation, in this case, ranges between 185kW and 271kW according to Fig 4. According to Fig 6, case 1b experienced a 1.64kg reduction in GHG. The improvement in the environmental safety is due to a decrease in load. The EENS, LOLE and LOLP, as indicated in Table 11, are not affected by the implementation of the DR program in this case. The power saved due to the introduction of DR, according to Fig 6, is not affected by energy source. However, the incentives paid to consumers on the DR program, according to Fig 10, are higher when renewable energy is part of the generation source of electricity.

The inclusion of renewable energy in the 20% energy from DG caused a 1.69% reduction in the transferred energy from the external grid compared to a 2.43% reduction when DG energy depends on a diesel generator. The non-stable nature of electricity from renewable sources caused a higher dependence on the external grid’s energy. Table 10, indicates that the running cost in case (1c), reduced by 62.92% compared with the base case. According to Fig 2, power losses in case 1c, reduce by 36.43%. Also, the integration of renewable energy into the distribution network caused the GHG emission reduced by 21.24%, according to Fig 6. Referring to Table 11, the EENS reduced by 43.65% while the LOLE and LOLP were decreased by 66.67%. The introduction of the renewable energies caused the utility benefit to decline by about 76.96%. The load profile, as displayed by Fig 4, shows load variation between about 196kW to 271kW. The peak load demand under case 1b and 1c reduced by about 4.9% of the base case, 1a.

According to Table 12, the total energy generation and the accompanying cost reduces as the importance attached to the cost of electricity increases. The reduction in energy generation causes an increase in the level of expected energy not supplied. Table 12 also shows that the incentives received by consumers. In Table 14, the maximum power is generated with the corresponding cost when the consumer power limitation is CM2. The power also corresponds to the minimum incentive to consumers.

### 5.2 Case 2

The implementation of DR in the presence of only diesel generator caused the total energy supply, according to Table 9, to reduce by about 8.28%. According to Table 10, the entire operation cost decrease by only 0.07%. Reference to Fig 3 shows that the technical losses experienced in the network under case 2b, declined by 8.7%. As indicated in Fig 5, the load profile varied between 1,721.708kW and 2,596.146kW, indicating about 8.9% reduction in the peak load demand. The GHG emission reduced according to Fig 4 by about 7%. The reliability indices, EENS, LOLE and LOLP recorded in Table 11 reduce to zero (0) representing 100% reliability.

The introduction of energy from wind and solar energy sources contributed to reducing the operation cost according to Table 10 by 72.32%. However, the table showed a reduction in utility benefit compared with that of case 2b. The introduction of renewable sources caused a 63.75% reduction in the electricity generated from diesel, according to Table 9. The 63.75% reduction in energy from a diesel generator, according to Fig 7, contributes to a 27.26% reduction in GHG emission. The values presented in Table 11 indicate that the EENS reduced by 62.33% while the LOLP and LOLP reduced by 33.33%.

Increased importance attached to the cost of generation according to Table 15, causes the energy generation and corresponding cost to reduce. However, the EENS increases with increase importance attached to generation. According to Table 15, as the limit of power curtailed by consumers (CM) increases, the generated energy and corresponding cost reductions. The reliability of the network, on the other hand, increases with an increase in CM.

## 6. Conclusion

The consideration of a domestic load profile showed that the operating cost of electricity reduced from GHȻ1,946,794 (when the DG energy is from only a diesel generator) to GH¢721,774.58 (when the DG energy comes from a diesel generator and renewable energies). Renewable energy contributed to 21.24% improvement in environmental safety. The reduction is also attributed to DR implementation. Combination of renewable energy and DR contributed to 36.43% reliability improvement. The peak load also reduced by 4.9%.

The introduction of renewable energy into the rural industrial network plus the implementation of CM considered for this study indicates a reduction in operating cost by 72.32%. The combination also caused the technical loss to reduce by 10.9% and GHG emission by 27.26%. The expected energy not supplied to consumers also reduced from 252.64kWh to 95.15kWh. Additionally, the peak load in this case reduced by 8.9%.

The implementation of DR in a distribution network positively influences the network operation cost, GHG emission, power loss, network reliability and load profile. The magnitude of influence, however, depends on the energy sources. The inclusion of renewable energy in the electricity generation, have the upper hand over total dependence on non-renewable energy sources, like the diesel. The running cost of a distribution network significantly reduces when renewable energy sources are part of electricity generation sources rather than non-renewable energy sources. However, the utility benefit reduces when renewable energy sources are part of the electricity generation sources. Therefore, the stress of ensuring a balance between supply and demand decreases with the implementation of DR.

Both utility companies and consumers stand to benefit from implementing the proposed DR program. The financial reward to the utility companies serves as a source of funds to further improve the existing network performance. On the other hand, electricity consumers can take advantage of the period for preventive maintenance or invest the money into other areas. An extra benefit for the consumers is direct the incentives received into generating electricity from other sources.

The implementation of DR is, therefore, worth considering in the Ghana distribution network. The move will promote the government of Ghana’s one district, one factory plan, towards the country’s economic advancement.

## Supporting information

S1 Appendix Layout of distribution network under consideration. (DOCX)

S2 Appendix Distribution network active power loss with and without DG. (DOCX)
