# Supplementary material for: Assessing the impact of demand response programs on the reliability of the Ghanian distribution network
Source: PLoS One. 2021 Mar 11;16(3):e0248012. doi: 10.1371/journal.pone.0248012 (PMC7951901; doi:10.1371/journal.pone.0248012)
Supplement: S2 Appendix — (DOCX) [file pone.0248012.s002.docx]

**Appendix 2**

**Distribution network active power loss with and without DG**
